# Projected Climate Vulnerability of *Salix babylonica* in China: Implications for Climate-Adaptive Urban Forestry

**DOI:** 10.3390/plants15152269

**Published:** 2026-07-24

**Authors:** Chunlei Yue, Xiaodeng Shi, Shiming Cheng

**Affiliations:** Zhejiang Academy of Forestry, Hangzhou 310023, China

**Keywords:** *Salix babylonica*, climate change, MaxEnt model, urban forestry, climate adaptation

## Abstract

Climate change is increasingly reshaping the distribution and long-term persistence of urban greening tree species, yet national-scale assessments of widely planted species in China remain limited. *Salix babylonica* is one of the most representative and extensively planted urban greening trees in China, but its future climate vulnerability and redistribution patterns are still poorly understood. In this study, we integrated 425 occurrence records and 16 environmental variables to project the potential distribution of *S. babylonica* under current and future (2050s, 2070s, and 2090s) climate scenarios across three shared socioeconomic pathways (SSP126, SSP370, and SSP585) using an optimized MaxEnt model. The optimized model demonstrated strong predictive performance and identified annual precipitation (≥309.64 mm), precipitation of the wettest month (≥82.01 mm), elevation (≤2251.24 m), mean temperature of the coldest quarter (≥−10.77 °C), annual mean temperature (≥3.94 °C), and minimum temperature of the coldest month (≥−19.11 °C) as the dominant environmental factors (threshold) constraining species distribution. Under the current climate, suitable habitats of *S. babylonica* are primarily distributed in East, Central, Southwest, and South China, with highly suitable habitats concentrated in the eastern and central plains. Under future climate scenarios, high suitability areas are projected to contract significantly and become progressively fragmented, with reductions ranging from 2.81% (2070s-SSP126) to 40.01% (2090s-SSP585), whereas medium and low suitability areas are projected to expand substantially, with increases ranging from 6.76% to 48.10%. The overall centroid of suitable habitats exhibits a consistent northward shift across scenarios. These results indicate that climate change will not simply expand the potential distribution of *S. babylonica*, but will reorganize habitat quality and increase long-term risks for its continued use in urban greening. Our findings provide a spatially explicit basis for climate-adaptive urban forestry planning and support risk-differentiated management, including the conservation of stable core areas, monitoring of declining risk zones, and cautious introduction trials in newly emerging suitable areas.

## 1. Introduction

Climate change is increasingly reshaping the performance, distribution, and long-term persistence of urban tree species [1]. As key components of urban green infrastructure, trees deliver essential ecosystem services, including regulating urban microclimate, carbon sequestration, air purification, and improvements in human well-being [2,3]. However, rising temperatures, changing precipitation regimes, and the increasing frequency of climatic extremes are altering the performance and distribution of many urban tree species [4]. Evidence based on nearly 1.2 million trees across 22 European cities indicates that, under a high-emission scenario, more than 80% of urban trees may experience hydrothermal conditions that constrain tree growth by 2070, with the proportion reaching almost 100% in cold semi-arid regions [5]. Similarly, a recent assessment in Lisbon projected that up to 77% of the distribution areas of Palearctic tree species in urban gardens may be exposed to high climate risk under future warming [6]. These findings suggest that many currently widespread urban tree species may have insufficient adaptive capacity to future climatic conditions, posing substantial challenges for the sustainability of urban greening.

This issue is particularly relevant in China, where rapid urbanization is occurring simultaneously with pronounced climate change [7]. A survey of more than 200,000 street trees across 11 major Chinese cities identified a significant negative association between tree health and latitude, with higher proportions of dead or declining trees occurring in colder, higher-latitude cities [8]. Furthermore, the effects of climate change on urban trees extend beyond gradual warming, but may also involve complex and sometimes unexpected ecological responses [9]. For example, warmer winters induced by climate change may accelerate spring phenology, while vulnerable young tissues remain vulnerable to episodic late frosts [10,11]. A recent projection covering 147 Chinese cities and 50 urban tree species further suggested that by the 2070s, 68% of species may experience a decline in suitable habitat area together with substantial range redistribution [12]. A passive response would incur multidimensional losses across ecological, economic, and social domains [13]. These studies collectively indicate that climate change is already reshaping the composition, health, and potential distribution of urban tree species in China, and highlight the need for species-specific assessments that can support climate-adaptive urban forestry planning.

Species distribution models (SDMs) provide an effective framework for characterizing species–environment relationships and projecting potential range shifts under future climate scenarios [14]. Among them, the MaxEnt model has been widely adopted owing to its robust performance with presence-only data and its capacity to capture complex environmental responses [15]. Previous studies have extensively applied MaxEnt to predict future distribution of several tree species in China, including *Magnolia denudata*, *Magnolia liliiflora* [16], *Robinia pseudoacacia* [17], and *Styphnolobium japonicum* [18], demonstrating its value for climate-risk screening and spatial planning. Nevertheless, for many ecologically important and widely planted urban greening species, national-scale and multi-scenario assessments remain scarce. This gap is especially important in urban forestry, where planting decisions often rely on current suitability, while future climate risks may be substantially underestimated.

*Salix babylonica* is one of the most iconic and widely planted urban greening tree species in China [19]. Native to East Asia and naturally distributed in the Yangtze and Yellow River basins, it is valued for its rapid growth, extensive root system, graceful architecture, and tolerance of waterlogging [20]. These characteristics have made it one of the preferred tree species for riverbank stabilization, urban parks, roadsides, and residential landscapes throughout China [21]. In addition to its ornamental and shading value, *S. babylonica* provides a wide range of ecosystem services such as carbon sequestration, water conservation, protection against erosion, and air purification [22]. A survey of street trees across 11 major Chinese cities identified Salix species (including *S. babylonica* and *S. matsudana*) as among the most dominant taxa in urban greening [8]. Among 65 common urban tree species in Wuhan, *S. babylonica* ranks among the top performers in carbon sequestration and oxygen release, with whole-plant rates of 288.93 g·d^−1^ and 209.80 g·d^−1^, respectively [23]. Given these ecological benefits, extensive planting of *S. babylonica* in cities offers considerable ecological and practical benefits for improving urban ecological environments, enhancing cityscapes, and elevating overall landscape quality. In addition, *S. babylonica* possesses notable pharmacological potential: extracts from its leaves, bark, and inflorescences have demonstrated a broad spectrum of therapeutic activities, including antithrombotic, antihypertensive, anti-inflammatory, analgesic, diuretic, hemostatic, immunomodulatory, anti-stress, and anti-tumor effects [24,25].

Despite its importance, research on *S. babylonica* under climate change has mainly focused on phenology, drought physiology, volatile organic compounds, and local environmental effects [26,27,28,29], whereas systematic assessments of its future climatic vulnerability and potential redistribution remain scarce. This knowledge gap is ecologically consequential, as phenological records indicate that the species’ flowering date has advanced significantly over the past 40 years due to winter warming—a trend that, when combined with extreme low-temperature events in early spring, has increased the incidence of foliar frost damage in temperate zones [30]. Such evidence suggests that future climate change may substantially alter both the performance and spatial suitability of this species in urban landscapes.

Despite its ecological and practical importance, a comprehensive understanding of how future climate change may reshape the potential distribution of *S. babylonica* remains lacking. Accordingly, this study pursued three specific objectives: (1) determine the dominant environmental factors constraining the distribution of *S. babylonica*; (2) quantify its current potential distribution and habitat suitability pattern; and (3) project future shifts in suitable habitats under multiple climate scenarios and discuss their implications for urban forestry planning and management. By integrating nationwide climate suitability modelling with practical urban forestry applications, this study provides a scientific basis for climate-adaptive tree selection, spatial optimization of urban greening, and risk-informed management strategies under future climate change.

## 2. Results

### 2.1. Model Optimization and Accuracy Assessment

When the MaxEnt model was run with default parameters (feature combination = LQHPT, regularization multiplier = 1), the avg.diff.AUC was 0.0463, the delta.AICc was 312.5127, the average 10% training omission rate (OR_10_) was 0.1666, and the avg.test.AUC was 0.7959 (Table 1). After parameter optimization using the ENMeval package, the optimal configuration was identified as feature combination LQH with a regularization multiplier of 4. Under these optimized settings, the avg.diff.AUC and average OR_10_ decreased by 61.99% and 31.21%, respectively, whereas the delta.AICc dropped to 0, and the average test AUC increased substantially. These improvements indicate that model overfitting was reduced by 61.99%, model complexity was markedly alleviated, and transferability was enhanced.

Using the optimized parameter settings, the MaxEnt model was run with ten replicate simulations. The mean training AUC and mean test AUC were 0.8337 and 0.8208, respectively—both exceeding 0.8 and substantially higher than the random expectation (AUC = 0.5). The Kappa coefficient was 0.7023, indicating good predictive performance and substantial agreement between predicted and observed distributions. Collectively, these metrics demonstrate that the optimized MaxEnt model achieved robust and reliable predictions of suitable habitats for *S. babylonica*.

### 2.2. Evaluation of Environmental Variables

The percent contribution and permutation importance of the 16 environmental variables used in the final model are presented in Table 2. The six most influential factors were annual precipitation (bio12), precipitation of the wettest month (bio13), elevation, mean temperature of the coldest quarter (bio11), annual mean temperature (bio1), and min temperature of the coldest month (bio6). Collectively, these six variables accounted for 90.0% of the percent contribution and 90.2% of the permutation importance, identifying them as the dominant environmental factors shaping the distribution of *S. babylonica*.

**Figure 1:**
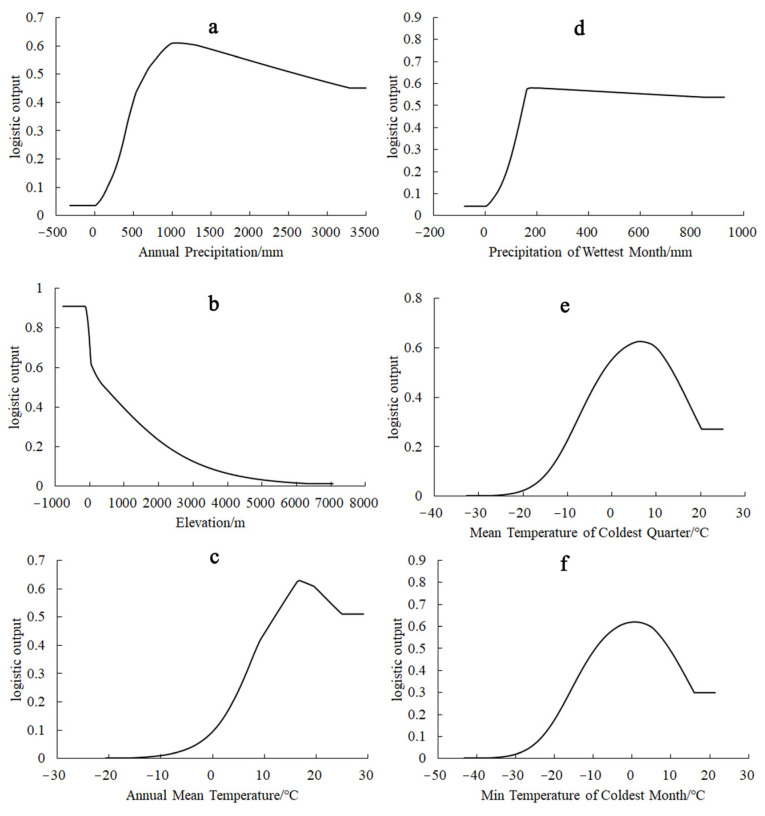
Response curves for the six dominant environmental variables. (**a**) Annual precipitation; (**b**) Elevation; (**c**) Annual Mean Temperature; (**d**) Precipitation of the wettest month; (**e**) Mean Temperature of Coldest Quarter; (**f**) Min Temperature of Coldest Month.

Response curves describing the relationships between the six dominant environmental variables and the occurrence probability of *S. babylonica* are presented in Figure 1. With increasing annual precipitation (bio12), precipitation of the wettest month (bio13), mean temperature of coldest quarter (bio11), annual mean temperature (bio1), and min temperature of coldest month (bio6), the occurrence probability exhibited a unimodal response—initially rising, peaking, and then gradually stabilizing. In contrast, the occurrence probability declined continuously with increasing elevation. Using the suitability threshold of 0.2 to define presence, the optimal environmental ranges for *S. babylonica* were identified as: bio12 ≥ 309.64 mm, bio13 ≥ 82.01 mm, elevation ≤ 2251.24 m, bio11 ≥ −10.77 °C, bio1 ≥ 3.94 °C, and bio6 ≥ −19.11 °C. These thresholds delineate the fundamental climatic and topographic constraints on the species’ distribution.

### 2.3. Current Potential Distribution Pattern

**Figure 2:**
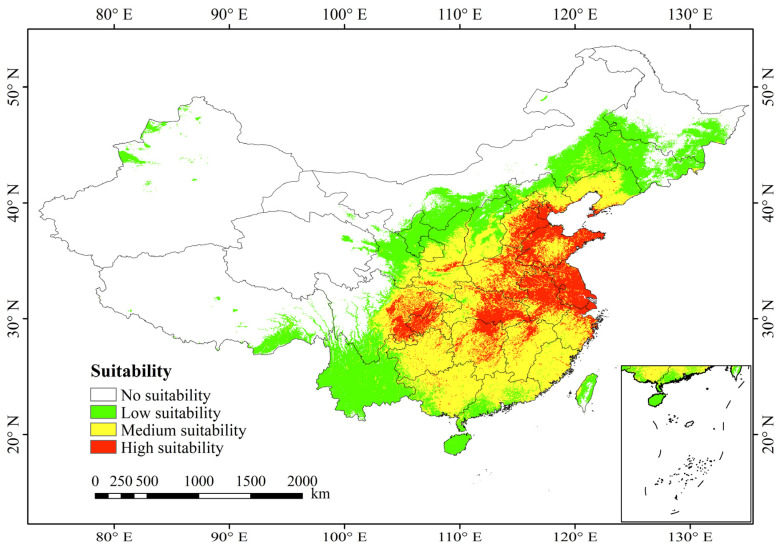
Current habitat suitability of *S. babylonica* in China.

Under current climatic conditions, the potentially suitable habitats of *S. babylonica* in China are widely distributed across parts of East, Central, Northeast, North, Northwest, Southwest, and South China (Figure 2), covering a total area of 411.86 × 10^4^ km^2^ (Table 3), which accounts for approximately 42.89% of the national land area. Suitable areas are predominantly located in southern China, with relatively limited distribution in North and Northeast China. High suitability areas (*p* ≥ 0.6) are mainly concentrated in the eastern and central plains, including eastern Sichuan, northern Hunan, most of Hubei, northern Jiangxi, northern Zhejiang, Jiangsu, Anhui, Shandong, most of Henan, southern Hebei, Beijing, Tianjin, and Shanghai. The total area of high suitability zones is 71.52 × 10^4^ km^2^, representing 7.45% of the study area. Medium suitability areas (0.4 ≤ *p* < 0.6) are primarily found in Guizhou, Guangxi, Guangdong, Fujian, most of Jiangxi, southern Zhejiang, western Hubei, southern Hunan, western Chongqing, southern Shaanxi, southern Shanxi, western Hebei, and southern Liaoning. These areas cover 181.53 × 10^4^ km^2^, accounting for 18.90% of the study area. Low suitability areas (0.2 ≤ *p* < 0.4) include Yunnan, southern Tibet, Hainan, Taiwan, southern Gansu, Ningxia, southeastern Inner Mongolia, northern Shaanxi, northern Shanxi, northern Hebei, northern Liaoning, southern Jilin, and southern Heilongjiang. Sporadic low suitability patches also occur in western Xinjiang (where medium suitability areas are also present). The total area of low suitability zones is 158.81 × 10^4^ km^2^, comprising 16.53% of the study area. The remaining areas, classified as unsuitable areas (*p* < 0.2), cover 548.62 × 10^4^ km^2^, accounting for 57.12% of the study area.

### 2.4. Future Potential Distribution Patterns

**Figure 3:**
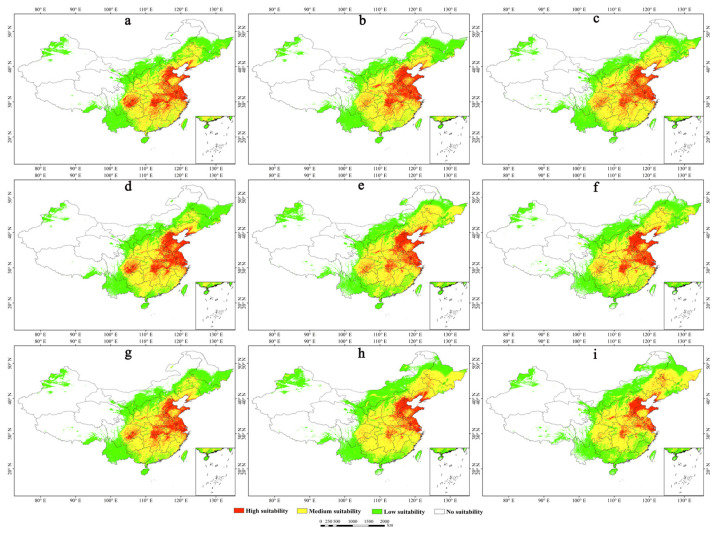
Projected habitat suitability of *S. babylonica* under future climate scenarios. (**a**) 2050s-SSP126; (**b**) 2050s-SSP370; (**c**) 2050s-SSP585; (**d**) 2070s-SSP126; (**e**) 2070s-SSP370; (**f**) 2070s-SSP585; (**g**) 2090s-SSP126; (**h**) 2090s-SSP370; (**i**) 2090s-SSP585.

**Figure 4:**
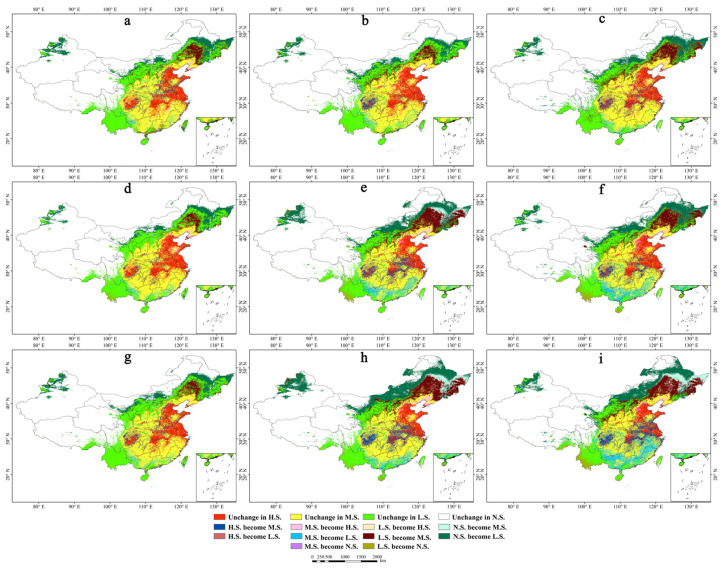
Changes in the habitat suitability area of *S. babylonica* under different climate scenarios. (**a**) 2050s-SSP126; (**b**) 2050s-SSP370; (**c**) 2050s-SSP585; (**d**) 2070s-SSP126; (**e**) 2070s-SSP370; (**f**) 2070s-SSP585; (**g**) 2090s-SSP126; (**h**) 2090s-SSP370; (**i**) 2090s-SSP585.

Following the same classification criteria, the potential distribution of *S. babylonica* was projected for three future periods (2050s, 2070s, and 2090s) under three SSP scenarios (SSP126, SSP370, and SSP585). The resulting spatial patterns, dynamic changes in suitability classes, and corresponding area statistics are presented in Figure 3 and Figure 4 and Table 3. Overall, the projected suitable habitats of *S. babylonica* exhibited substantial shifts in both location and extent across scenarios and time horizons. A general trend of contraction was observed for high suitability areas and unsuitable areas, whereas medium and low suitability areas expanded considerably.

High suitability areas showed a declining trend under all scenarios, with reductions ranging from 2.81% to 40.01% relative to the current baseline. Under SSP126, the area initially decreased, experienced a slight recovery, and then declined again. Under SSP370 and SSP585, high suitability areas decreased monotonically over time, accompanied by increased fragmentation. The most severe contraction occurred under SSP585 in the 2090s, with a high suitability area reduced to 42.90 × 10^4^ km^2^—a 40.01% decrease from the present. This loss was primarily concentrated in eastern Sichuan, southern Hunan, Anhui, most of Jiangsu, and southern Shandong, where high suitability areas are projected to transition to medium suitability.

Medium suitability areas exhibited a consistent expansion trend across all scenarios. Under SSP126 and SSP585, the increase first accelerated and then slowed, while under SSP370, the expansion rate continuously intensified. Overall, the increase ranged from 6.76% to 48.10%. The largest expansion occurred under SSP370 in the 2090s, reaching 268.85 × 10^4^ km^2^. This gain was primarily attributable to the conversion of former high suitability zones, as well as to upgrades from low suitability areas in eastern Inner Mongolia, eastern Heilongjiang, southern Jilin, and northern Liaoning.

Low suitability areas also increased across all future scenarios, with the magnitude of expansion growing progressively over time. The overall increase ranged from 17.46% to 36.87%. The maximum extent was projected under SSP370 in the 2090s, reaching 217.36 × 10^4^ km^2^. This expansion was largely driven by the conversion of previously unsuitable lands in northern Heilongjiang, central and eastern Inner Mongolia, northern Jilin, and western Xinjiang.

Unsuitable areas declined steadily with increasing radiative forcing and over time, with reductions ranging from 7.83% to 22.75% across scenarios.

### 2.5. Centroid Shift of Potential Suitable Areas

**Figure 5:**
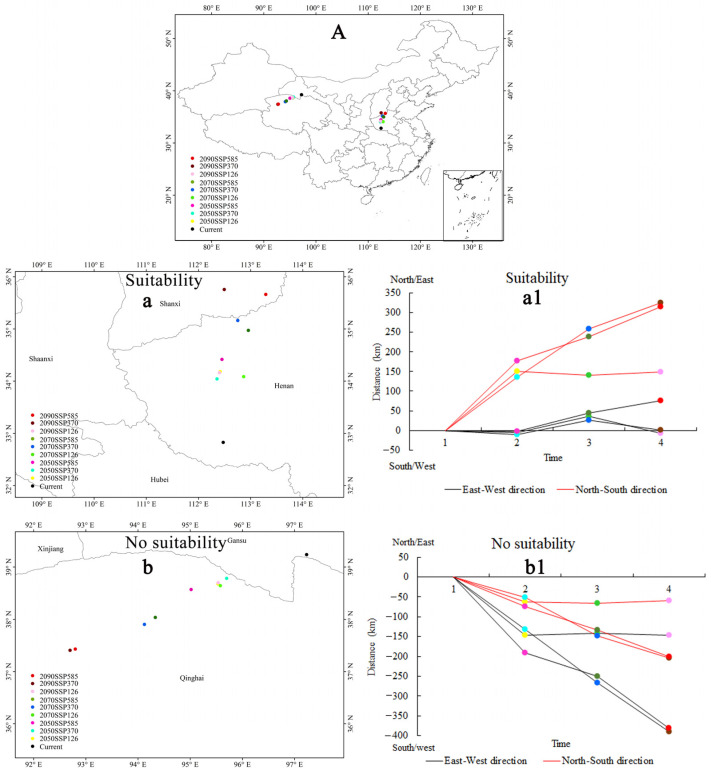
Migration of the centroid of *S. babylonica* under different conditions. (**A**) Centroids in China; (**a**) Centroids in suitable area. (**b**) Centroids in unsuitable area; (**a1**,**b1**) indicates the distances that centroids of different grades of suitable distribution area migrate in two directions (north–south and east–west) under future climate change.

As illustrated in Figure 5, the centroid of the potentially suitable distribution of *S. babylonica* under future climate scenarios was consistently located within Henan Province. Across all scenarios and time periods, the centroid exhibited a clear and consistent northward shift, indicating progressive poleward migration of suitable habitats in response to future climate change.

## 3. Discussion

### 3.1. Key Environmental Constraints on Distribution

This study represents the first national-scale attempt to integrate multi-source occurrence data with an optimized MaxEnt modeling framework to systematically simulate and project the potential distribution patterns of the urban greening species *S. babylonica* under both current and future climate scenarios. Our results identified annual precipitation (bio12), precipitation of the wettest month (bio13), elevation, mean temperature of the coldest quarter (bio11), annual mean temperature (bio1), and min temperature of the coldest month (bio6) as the dominant environmental factors shaping the distribution of *S. babylonica*, confirming that its suitable habitats are primarily governed by the synergistic interplay of water and thermal regimes—a finding broadly consistent with the climatic determinism paradigm in plant biogeography [31]. Zhang et al. [32] identified temperature and precipitation as the primary environmental factors influencing the distribution of *Anoplophora glabripennis* and its host plant *S. babylonica*, with precipitation of the wettest month and minimum temperature of the coldest month being the most important variables shaping the distribution of *S. babylonica*—a finding that is broadly consistent with our results. These parallels validate the robustness of our modeling approach and confirm that our findings on the declining suitability of *S. babylonica* are not idiosyncratic but reflect a broader trend affecting multiple Salix species in China.

Notably, precipitation-related factors (bio12 and bio13) together contributed 45.8% of the model’s predictive power, establishing water availability as the single most critical constraint on the species’ geographical distribution and underscoring the unique ecological niche of *S. babylonica* as a hygrophilous, widespread riparian tree [21]. The predominant role of annual precipitation (bio12, 24.46%) aligns with previous MaxEnt-based studies on temperate urban greening tree species such as *Fraxinus mandshurica* [33] and *Styphnolobium japonicum* [18]. The response threshold derived for bio12 (≥309.64 mm) corresponds closely to the species’ intrinsic biological traits: as a typical hygrophilous, watercourse-associated tree, *S. babylonica* possesses an extensive root system and sustains high transpiration rates, rendering it heavily dependent on adequate soil moisture [20]. Our macroscale results thus provide quantitative corroboration of earlier regional observations that the distribution of *S. babylonica* in arid and semi-arid zones is strictly confined to oasis corridors and riverine margins [19]. Of particular ecological significance is the markedly high contribution of precipitation during the wettest month (bio13, 16.77%), which ranked second among all predictors. It suggests that water supply during the critical growth season, rather than the evenness of mean annual precipitation, is the more decisive determinant of the species’ competitive establishment and vigorous performance [34]. Ample summer precipitation is directly coupled with peak photosynthetic carbon gain and physiological metabolism in *S. babylonica*, a species characterized by high transpiration demand [35]. This pronounced sensitivity to growing-season water availability is corroborated by physiological evidence: under drought stress, chlorophyll a, chlorophyll b, and total chlorophyll contents in *S. babylonica* decline, while the activities of protective enzymes (SOD, POD, CAT) increase and osmotic adjustment substances (soluble protein, soluble sugar, and proline) accumulate—responses that collectively underscore the species’ limited intrinsic drought tolerance [36]. Comparative physiological studies on *S. babylonica* and the more drought-tolerant *S. matsudana* have shown that while *S. babylonica* exhibits higher photosynthesis, stomatal conductance, and stem hydraulic conductivity, it possesses lower water use efficiency and hydraulic safety, indicating a trait-based trade-off that prioritizes rapid gas exchange over water conservation [37].

Winter low temperature represents the primary bottleneck constraining the northward distribution of *S. babylonica*. Both mean temperature of the coldest quarter (bio11) and min temperature of the coldest month (bio6) were identified as key limiting variables, with their respective suitability thresholds (≥−10.77 °C and ≥−19.11 °C) delineating the northern boundary of the species’ potential range. This threshold range falls within the species’ physiological tolerance limit, as the semi-lethal temperature (LT_50_) of *S. babylonica* was determined to be −28.18 °C [38]. Low-temperature stress triggers an initial elevation of SOD and POD activities, soluble protein, proline, and MDA content in *S. babylonica*; however, these indicators decline when the stress exceeds the semi-lethal temperature (LT_50_), suggesting that the species’ cold tolerance has a critical physiological threshold [38]. Extreme low temperatures can trigger intercellular ice formation and protoplast dehydration, leading to irreversible xylem embolism and cambial damage, thereby inhibiting plant growth and development [39]. This physiological constraint is supported by national-scale phenological evidence, which indicates that *S. babylonica* experiences considerable spring leaf frost damage, with the most severe damage concentrated north of 50° N and in the western Qinghai–Tibet Plateau [29]. By quantifying these critical temperature thresholds, our study provides essential parameters for predicting the species’ potential poleward expansion under climate warming scenarios. Nevertheless, the substantial contribution of annual mean temperature (bio1, 10.08%) suggests that the distribution of *S. babylonica* is not governed solely by extreme cold events, but rather reflects the integrated effect of annual thermal conditions. Excessively low annual mean temperatures can still constrain photosynthetic capacity and biomass accumulation, even in the absence of acute freezing stress [40].

Elevation also exerts a strong regulatory influence on the potential distribution of *S. babylonica*. This effect is largely indirect, primarily mediated by elevation-driven changes in local thermal and hydrological regimes [41]. Specifically, decreasing temperatures (approximately 0.6 °C per 100 m increase) and alterations in orographic precipitation patterns reshape the local hydrothermal combination that governs tree growth [42]. Our results show that the occurrence probability of *S. babylonica* declines monotonically with increasing elevation, with an upper suitability limit of approximately 2251 m. Although a documented seedling nursery for this species has been reported at 2476 m in Gonghe County, Qinghai Province [43], our modeled threshold is more conservative, lending credence to its reliability. This pattern is consistent with the general principles of elevational zonation in mountain ecosystems, where persistent low temperatures, intensified solar radiation, and abbreviated growing seasons collectively impose fundamental physiological constraints on tree survival and establishment at higher elevations [44].

The relative importance of environmental factors may not be uniform across the species’ entire geographic range. At the northern (cold) edge of the distribution, temperature-related variables—particularly min temperature of the coldest month (bio6) and mean temperature of the coldest quarter (bio11)—are likely to exert stronger constraints on survival, as freezing stress becomes increasingly limiting. Conversely, at the southern or arid margins, precipitation-related factors (bio12, bio13) may become more dominant, as water availability increasingly limits establishment and growth. This spatial heterogeneity in limiting factors implies that climate change may drive distributional shifts differently at different range edges: warming may alleviate cold constraints at the northern edge, potentially facilitating poleward expansion, while increasing drought stress at the southern or arid edges may accelerate habitat contraction.

### 3.2. Climate-Driven Redistribution and Reorganization of Habitat Quality

Comparing the projected future distributions of *S. babylonica* with its current potential range reveals a complex pattern characterized by northward expansion of overall suitable areas yet pronounced contraction of high-quality habitats. This pattern is consistent with recent findings from related studies. Zhang et al. [32] projected that under future climate change, the suitable range of *S. babylonica*—the host plant of *Anoplophora glabripennis*—would expand and shift northward. Gu et al. [12] examined 50 city tree species across 147 Chinese cities and found that 68% of species are projected to experience a contraction of suitable habitat area under future climate scenarios. Similarly, Gao et al. [17], using an optimized MaxEnt model to predict the spatial distribution of *Robinia pseudoacacia*—another major urban greening species in China—reported that while the total suitable area remained largely consistent with current conditions, the high-suitability area decreased and the overall distribution shifted northeastward. These comparative analyses suggest that the dual pattern of northward expansion and high-suitability contraction observed for *S. babylonica* may reflect a broader response trend among temperate urban tree species in China. This duality encapsulates the multifaceted nature of climate change impacts on tree species, reflecting both opportunities and escalating risks.

On one hand, climate warming is expected to alleviate cold stress, thereby facilitating poleward range expansion [45]. Our simulations consistently show a northward shift of the species’ distribution centroid by the end of this century, with notable expansion of medium- and low-suitability areas in eastern Inner Mongolia, Heilongjiang, and northern Xinjiang. This trend aligns with a substantial body of evidence documenting poleward and upslope migrations of temperate tree species in response to rising temperatures, and is consistent with the fundamental ecological principle that species track their climatic niches [46]. The northward shift was most pronounced under the high-emission scenario (SSP585), where thermal constraints are most relaxed.

On the other hand, and contrary to the simple expectation that warming uniformly benefits temperate trees, our projections reveal a significant and consistent decline in high suitability areas—the most stable and ecologically optimal habitats. Under SSP585, high-suitability zones are projected to contract by 40.01% by the 2090s. This counterintuitive outcome strongly suggests that increased temperature alone does not guarantee improved habitat quality; rather, it may be accompanied by a suite of intensifying stressors that collectively undermine the species’ performance and competitive advantage [47].

Three interrelated mechanisms may explain this phenomenon. First, hydrothermal decoupling could play a key role. Although annual precipitation is projected to increase in many regions under future scenarios, the seasonal distribution of rainfall is expected to become more uneven, with heightened interannual variability and more frequent extremes—both prolonged droughts and intense deluges [48]. While *S. babylonica* is tolerant of waterlogged conditions, it is sensitive to soil moisture deficits [36]. Chronic or periodic water stress during the growing season can severely suppress photosynthesis and growth, preventing populations in newly expanded areas from attaining high-suitability status. Second, phenological mismatch and late-spring frost damage represent an additional risk. Warmer winters induced by climate change have advanced the onset of spring phenology—such as budburst and leaf expansion—in *S. babylonica*, as documented by previous phenological records [29]. However, the frequency and intensity of extreme low-temperature events in early spring have not decreased correspondingly [49]. This temporal coupling of early leaf emergence followed by episodic frost renders vulnerable new tissues highly susceptible to irreversible freezing injury, a mechanism identified as a key driver of damage and mortality in many temperate tree species [30]. Our distribution-level risk assessment provides macroscale support for its significance in *S. babylonica*. Third, the increasing intensity and frequency of summer heatwaves may further constrain the species’ performance. Projected rises in extreme high temperatures during the warm season may approach or exceed the photosynthetic optimum of *S. babylonica* [28]. Physiological studies on Salix species indicate that, although they possess moderate thermotolerance, sustained exposure to supra-optimal temperatures can induce photoinhibition, membrane damage, and elevated respiratory carbon losses—collectively constraining growth and vigor even in the absence of lethal heat [36].

Taken together, the future distribution dynamics of *S. babylonica* represent the net outcome of a trade-off between warming-driven opportunities for range expansion and the increasing frequency and intensity of multiple, often covarying, climatic stressors. The pronounced contraction of high-suitability zones in the species’ current core range—including the North China Plain and the middle–lower Yangtze River Basin—exemplifies scenarios where escalating risks outweigh the benefits of thermal relaxation. For urban systems that heavily depend on the ecosystem services provided by *S. babylonica*, these projected shifts portend substantial challenges and underscore the urgency of integrating climate risk assessment into urban forest planning and management.

### 3.3. Implications for Climate-Adaptive Urban Forestry

Our findings provide spatially explicit, decision-relevant insights for enhancing climate adaptation and fine management in urban forestry. First, this study underscores the urgent need to shift urban tree species planning from a “current suitability” to a “future adaptation” paradigm. The long-term (30–50 years) survival risks of *S. babylonica* are substantially underestimated in many cities where it is currently planted as a highly suitable species. Urban planners and landscape managers should therefore consult the multi-temporal risk maps generated in this study to reassess the viability and expected service life of *S. babylonica* in major greening projects. In particular, large-scale new plantings should be avoided in cities projected to transition from high to medium or low suitability in the coming decades, thereby mitigating future “climate debt” [50]. Certainly, these thresholds provide city managers with a straightforward, data-driven tool for screening planting suitability: municipalities falling within the defined climatic envelopes (bio12 ≥ 309.64 mm, bio6 ≥ −19.11 °C, elevation ≤ 2251.24 m) can continue to utilize *S. babylonica* with confidence, whereas those outside these ranges should prioritize alternative species or implement adaptive management measures.

Second, we advocate for risk-zone-based, differentiated management strategies. In core conservation zones—areas identified as persistently highly suitable both currently and in the future—priority should be given to the in situ conservation of germplasm resources and landscape heritage. Enhanced health monitoring and maintenance of existing mature and ancient trees should be implemented to sustain their ecosystem service delivery [51]. In risk monitoring zones—areas currently highly suitable but projected to undergo significant future decline—adaptive management trials should be initiated proactively. These may include supplemental irrigation to alleviate drought stress, conversion to mixed stands to buffer local microclimatic extremes, and the introduction of locally adapted genetic materials with greater tolerance to heat and water deficit for enrichment planting [52]. In potential introduction zones—areas currently of low suitability but projected to become medium or highly suitable—cautious, small-scale field trials and long-term monitoring should be conducted, particularly in regions such as northern North China and southern Northeast China [53]. Such trials must be accompanied by rigorous assessments of local edaphic conditions, water availability, and extreme climate risks; large-scale, indiscriminate promotion should be strictly avoided.

It is also important to note that many ‘weeping willows’ cultivated outside China, particularly in Europe and North America, are actually hybrids such as *Salix* × *sepulcralis* (*S. alba* × *S. babylonica*), which exhibit considerably greater cold hardiness [54]. For cities in northern China or other regions projected to experience increased climatic stress, these cold-hardy hybrids may serve as viable alternatives to pure *S. babylonica*, providing similar ornamental value with enhanced resilience to harsh winter conditions. We therefore recommend that urban forestry authorities in marginal climatic zones consider pilot trials of *Salix × sepulcralis* cultivars as part of their climate-adaptive planting strategies.

Finally, we recommend that the vulnerability assessment of keystone tree species be formally integrated into urban forest biodiversity and resilience evaluation frameworks [55]. As a dominant species in many Chinese cities [56], the projected widespread decline of *S. babylonica* exemplifies the “single-species risk”—a scenario in which over-reliance on one or a few taxa creates functional vulnerabilities in critical ecosystem services such as shading, cooling, and aesthetic value. Should a large-scale decline occur, these services may face abrupt and prolonged disruption. Urban forestry authorities should therefore take this study as a precedent and systematically conduct analogous climate vulnerability assessments for other major greening species. Such efforts are essential for the evidence-based design of climate-resilient urban forests characterized by high species diversity, structural complexity, and functional redundancy. Reducing excessive dependence on a limited set of species is, ultimately, the foundational pathway toward the long-term sustainability of urban forestry under accelerating climate change.

### 3.4. Limitations and Future Research Directions

Despite the improved predictive accuracy achieved through model optimization, several limitations inherent in this study should be acknowledged, which also point toward promising avenues for future research. First, the MaxEnt algorithm assumes niche equilibrium and unconstrained dispersal—assumptions that are frequently violated in urban environments, where heterogeneous land cover, impervious surfaces, artificial irrigation, pollution, and active human management profoundly modify local hydrothermal regimes and render tree migration entirely dependent on anthropogenic planting [57]. Future studies should therefore integrate spatially explicit layers representing land use, urban heat island intensity, and anthropogenic interventions to develop urban-specific ecological niche models that more realistically simulate the fate of tree species in cities. Meanwhile, although MaxEnt is a well-validated and widely used algorithm for presence-only data, we acknowledge that comparing multiple species distribution models—for example, through ensemble approaches such as BIOMOD2—could further enhance methodological robustness [38]. Future studies could incorporate generalized linear models, random forests, and gradient boosting models alongside MaxEnt to assess whether ensemble predictions yield different conclusions regarding the climate vulnerability of *S. babylonica*.

Second, this study treated *S. babylonica* as a homogeneous taxonomic unit, yet populations distributed across distinct climatic gradients may have undergone local adaptation, resulting in genetic differentiation in key functional traits such as cold tolerance and drought resistance [58]. Neglecting such intraspecific variation risks either overestimating the species’ overall vulnerability or underestimating its adaptive capacity under future climates; integrating population genomics with common garden experiments to conduct genotype–environment association analyses represents a critical next step toward identifying and deploying provenances with superior climate resilience. Third, our modeling framework did not account for biotic interactions, including the climate-driven range expansion of pests and pathogens (e.g., *Anoplophora glabripennis*, *canker diseases*) or shifts in competitive dynamics with co-occurring native and invasive plant species [56]. These biotic stressors may act synergistically with climatic stresses, amplifying physiological damage and accelerating population decline [59]; developing multi-factor coupled models that integrate both abiotic and biotic drivers remains a substantial methodological frontier.

Finally, predicting habitat suitability addresses only whether a species “can survive,” but for urban forest management, understanding how well it will perform and whether its ecosystem functions will be sustained is equally critical [51]. Future efforts should therefore link species distribution models with process-based mechanistic models—such as tree growth models and coupled photosynthesis–transpiration models—to project climate-induced changes in key functional indicators, including biomass accumulation, carbon sequestration potential, and cooling efficiency, thereby providing direct, quantitative baselines for ecosystem service valuation under future scenarios.

It is important to acknowledge that our model was calibrated exclusively on occurrence data from China, despite *S. babylonica* being widely introduced and cultivated across Asia, Europe, and the Americas. China’s diverse climatic gradients—from cold temperate to subtropical and arid to humid—broadly represent the range of conditions found across much of the species’ global cultivated range. Our identified environmental conditions may therefore serve as reference points for assessing planting suitability in other regions worldwide, though local validation is recommended. Future studies could usefully compare regional and global MaxEnt models to assess whether global occurrence data reveal additional climatic tolerances not captured in the native range.

In sum, this study delineates the spatial patterns of climate-related risks facing *S. babylonica* and offers a science-based blueprint for proactive intervention. The convergence of macro-scale distribution modeling with micro-scale mechanistic understanding, and the integration of fundamental ecological principles with context-specific urban management practices, represent the essential pathway toward the development of future-oriented, resilient urban forest ecosystems.

## 4. Materials and Methods

**Figure 6:**
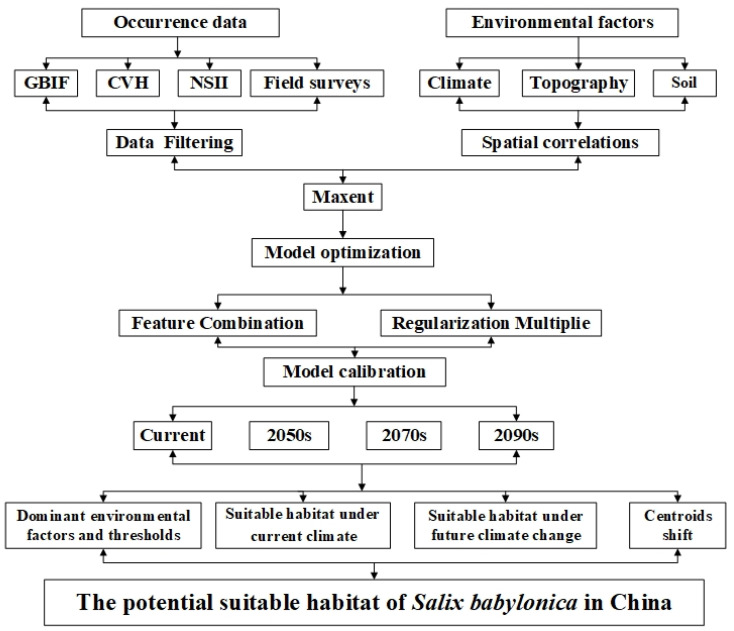
Flow chart for predicting potential suitable habitat.

The methodology of this study comprises five main steps: (1) compiling and processing occurrence records together with multi-source environmental variables; (2) screening environmental predictors via correlation analysis to reduce multicollinearity; (3) optimizing MaxEnt model parameters using the ENMeval package and calibrating the model with optimal settings; (4) projecting current and future habitat suitability under multiple climate scenarios; and (5) delineating suitability classes, calculating their areas, and analyzing centroid shifts to characterize distributional dynamics. The step-by-step workflow is illustrated in Figure 6.

### 4.1. Occurrence Data of S. babylonica

**Figure 7:**
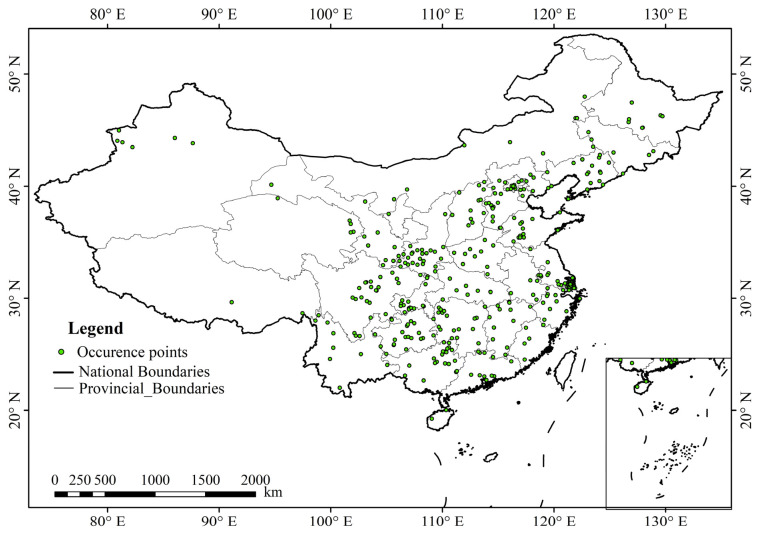
Occurrence records of *S. babylonica* in China.

Occurrence records of *S. babylonica* were compiled from multiple sources, including the Global Biodiversity Information Facility (GBIF, https://www.gbif.org, accessed on 16 July 2026), the Chinese Virtual Herbarium (CVH, www.cvh.ac.cn), the National Specimen Information Infrastructure (NSII, http://www.nsii.org.cn), and field surveys conducted from May 2022 to May 2023. A total of 527 occurrence points was initially collected. After removing records with missing or invalid spatial information and eliminating duplicates within individual grid cells, 425 spatially unique occurrence points were retained for model development (Figure 7).

### 4.2. Environmental Data

A set of 33 environmental variables encompassing climate, soil, and topography was initially compiled (Table S1). Climatic data, including the 19 bioclimatic variables and elevation, were obtained from the WorldClim version 2.1 database (https://www.worldclim.org/). Slope and aspect were derived from the elevation data (Table S1). Soil variables were obtained from the Harmonized World Soil Database (HWSD) (http://webarchive.iiasa.ac.at/Research/LUC/External-World-soil-database/HTML/index.html?sb=1, accessed on 10 December 2023). Temporal periods for climatic data included the present (1970–2000) and three future periods: 2050s (2041–2060), 2070s (2061–2080), and 2090s (2081–2100). All environmental datasets were acquired at a spatial resolution of 2.5 arc-min and projected to the WGS_1984 coordinate system. Future climate projections were derived from the BCC-CSM2-MR model developed by the Beijing Climate Center [60]. Three shared socioeconomic pathway (SSP) scenarios were considered: SSP126 (low emission), SSP370 (medium-high emission), and SSP585 (high emission).

Spatial correlations were observed among the environmental variables. The inclusion of highly correlated variables in species distribution modeling can increase model complexity, lead to overfitting, and reduce predictive performance [61]. Therefore, prior to final model calibration, Pearson correlation analysis was performed to assess collinearity among all candidate variables. For variable pairs with a correlation coefficient |r| ≥ 0.8, the variable considered to be more ecologically meaningful was retained, whereas the other was excluded. Following this procedure, 16 environmental variables were retained for subsequent modeling (Table S2).

### 4.3. Model Optimization

MaxEnt version 3.4.1 (http://biodiversityinformatics.amnh.org/open_source/MaxEnt/, accessed on 15 August 2022) was used to predict the potential distribution for *S. babylonica* in four periods [62]. Given the inherent complexity of the MaxEnt algorithm, the model is sensitive to sampling bias, and its predictive performance is strongly influenced by the choice of feature combination (FC) and regularization multiplier (RM) [63]. The available feature classes include linear (L), quadratic (Q), hinge (H), product (P), and threshold (T). In this study, we tested six feature combinations (L, LQ, H, LQH, LQHP, and LQHPT) and eight regularization multipliers (0.5, 1.0, 1.5, 2.0, 2.5, 3.0, 3.5, 4.0) using the “ENMeval” package in R [64]. Model performance was evaluated based on the Akaike Information Criterion corrected for small sample sizes (AICc), the delta AICc, the difference between the training and testing AUC values (AUC.DIFF), and the 10% training omission rate (OR_10_) [47].

### 4.4. Model Calibration

After determining the optimal feature combination and regularization multiplier, we configured the MaxEnt model accordingly. Occurrence records were randomly partitioned into training (75%) and testing (25%) datasets [65]. The remaining parameters were retained at their default settings. To reduce the influence of random data partitioning, we performed ten replicate runs for each scenario. The output format was set to ASCII with logistic output [66].

The importance of environmental variables was evaluated using percent contribution and permutation importance [67]. Model performance was assessed using the area under the receiver operating characteristic curve (AUC), which ranges from 0 to 1, with values closer to 1 indicating higher predictive accuracy [68].

### 4.5. Delineation of Suitable Distribution Areas

The ASCII output files generated by MaxEnt 3.4.1 were imported into ArcGIS 10.4 and converted into raster format. Suitable habitat classes were defined using the natural breaks classification method based on predicted occurrence probability (P) [68]: unsuitable areas (*p* < 0.2), low suitability areas (0.2 ≤ *p* < 0.4), medium suitability areas (0.4 ≤ *p* < 0.6), and high suitability areas (*p* ≥ 0.6). These habitat classes were visualized with different color schemes. The area of each suitability class was calculated based on the number of corresponding raster grid cells.

## 5. Conclusions

This study provides the first national-scale assessment of the impacts of future climate change on the potential distribution of *S. babylonica*, an iconic urban greening tree species in China. Our findings identify precipitation, elevation, and low temperature as the dominant environmental factors shaping the species’ distribution. Under current climatic conditions, the suitable areas for *S. babylonica* are mainly concentrated in East, Central, Southwest, and South China, as well as in parts of Northeast, North, and Northwest China. Future climate change is projected to drive a significant contraction and increased fragmentation of high suitability areas, while the overall suitable range will continue to shift northward and expand. These results indicate that climate change will not simply expand the distribution of *S. babylonica*, but will fundamentally reshape its spatial pattern, thereby increasing long-term risks associated with its use in urban greening. Based on these findings, we propose a three-tier spatial management framework for urban greening—comprising core conservation zones, risk monitoring zones, and potential introduction zones—to provide actionable decision support for climate-smart urban greening. This framework holds significant theoretical and practical value for advancing urban forestry from traditional experience-based management toward precise, science-driven, and forward-looking practices, and for ensuring the long-term stability of urban forest ecosystem services in the face of future climate uncertainty.

## Figures and Tables

**Table 1:** Evaluation of MaxEnt model with default and optimised parameter settings.

Settings	Regularization Multiplier	Feature Combination	avg.test.AUC	avg.diff.AUC	avg.OR_10_	delta.AICc
Default	1	LQHPT	0.7959	0.0463	0.1666	312.5127
Optimized	4	LQH	0.8069	0.0176	0.1146	0

**Table 2:** Contribution and permutation importance of selected environmental factors of *S.babylonica*.

Code	Description	Percent Contribution	Permutation Importance
bio12	Annual Precipitation	24.46	26.29
bio13	Precipitation of Wettest Month	16.77	7.85
elevation	Elevation	15.73	24.20
bio11	Mean Temperature of Coldest Quarter	13.18	22.65
bio1	Annual Mean Temperature	10.08	1.53
bio6	Min Temperature of Coldest Month	9.74	7.72
slope	Slope	1.80	0.32
bio7	Temperature Annual Range (bio5- bio6)	1.74	1.21
bio3	Isothermality (bio2/bio7) (* 100)	1.71	0.61
bio2	Mean Diurnal Range (Mean of monthly (max temp-min temp))	1.52	0.39
t-clay	Topsoil Clay Fraction	1.18	2.61
t-usda	Topsoil USDA Texture Classification	0.75	0.90
t-silt	Topsoil Silt Fraction	0.48	1.09
t-ph	Topsoil pH (H_2_O)	0.32	0.47
bio15	Precipitation Seasonality (Coefficient of Variation)	0.32	1.72
aspect	Aspect	0.22	0.44

**Table 3:** Changes in the suitable area of *S. babylonica* in different scenarios.

Scenario	Period	High Suitability Area		Medium Suitability Area		Low Suitability Area		Unsuitable Area	
		Area (×10^4^/km^2^)	Change	Area (×10^4^/km^2^)	Change	Area (×10^4^/km^2^)	Change	Area (×10^4^/km^2^)	Change
current		71.52		181.53		158.81		548.62	
SSP126	2050s	64.51	−9.80%	212.92	17.29%	190.82	20.16%	492.14	−10.29%
	2070s	69.51	−2.81%	193.80	6.76%	191.51	20.59%	505.63	−7.83%
	2090s	59.00	−17.50%	205.69	13.31%	203.79	28.32%	491.93	−10.33%
SSP370	2050s	66.81	−6.59%	207.72	14.43%	190.54	19.98%	495.33	−9.71%
	2070s	55.37	−22.58%	243.92	34.37%	197.59	24.42%	463.55	−15.50%
	2090s	50.43	−29.49%	268.85	48.10%	217.36	36.87%	423.78	−22.75%
SSP585	2050s	61.93	−13.41%	233.31	28.53%	186.53	17.46%	478.63	−12.75%
	2070s	58.75	−17.85%	215.25	18.58%	212.26	33.66%	474.15	−13.57%
	2090s	42.90	−40.01%	253.86	39.85%	216.93	36.60%	446.71	−18.57%

## Data Availability

The original contributions presented in this study are included in the article/Supplementary Material. Further inquiries can be directed to the corresponding authors.

## Supplementary Materials

The following supporting information can be downloaded at https://www.mdpi.com/article/10.3390/plants15152269/s1. Table S1: The environmental variables; Table S2: The environmental variables used in this study.
