# Supplementary material for: Projected Climate Vulnerability of Salix babylonica in China: Implications for Climate-Adaptive Urban Forestry
Source: Plants (Basel). 2026 Jul 24;15(15):2269. doi: 10.3390/plants15152269 (PMC13468232; doi:10.3390/plants15152269)
Supplement: Supplementary file 1 [file plants-15-02269-s001.zip › plants-4410806-supplementary.pdf]

Table S1. The Environmental variables

Type	Code	Description	Unit
Climate	bio1	Annual mean temperature	℃
	bio2	Mean diurnal range (Mean of monthly (max.temp.-min.temp.)	℃
	bio3	Isothermality (bio2 / bio7) (× 100)	
	bio4	Temperature seasonality (standard deviation*100)	
	bio5	Max temperature of the warmest month	℃
	bio6	Min temperature of the coldest month	℃
	bio7	Temperature annual range (bio5- bio6)	℃
	bio8	Mean temperature of the wettest quarter	℃
	bio9	Mean temperature of the driest quarter	℃
	bio10	Mean temperature of the warmest quarter	℃
	bio11	Mean temperature of the coldest quarter	℃
	bio12	Annual precipitation	mm
	bio13	Precipitation of the wettest month	mm
	bio14	Precipitation of the driest month	mm
	bio15	Precipitation seasonality (Coefficient of variation)	
	bio16	Precipitation of the wettest quarter	mm
	bio17	Precipitation of the driest quarter	mm
	bio18	Precipitation of the warmest quarter	mm
	bio19	Precipitation of coldest quarter	mm
Soil	t-pH	Topsoil pH (H_2O)	
	t-clay	Topsoil clay fraction	%
	t-bulk	Topsoil bulk density	kg/dm^3
	t-oc	Topsoil organic carbon	
	t-ece	Topsoil electric conductivity	
	t-esp	Topsoil Sodicity (ESP-exchangeable sodium percentage)	%
	t-gravel	Topsoil gravel content	%
	t-sand	Topsoil sand fraction	%
	t-silt	Topsoil silt fraction	%
	t-texture	Topsoil soil texture	
	t-usda	Topsoil USDA texture classification	
topographical	elevation	Elevation	m
	slope		°
	aspect		rad

Table S2 The environmental variables used in this study

Type	Code	Description	Unit
Climate	bio1	Annual mean temperature	℃
	bio2	Mean diurnal range (Mean of monthly (max.temp.-min.temp.)	℃
	bio3	Isothermality (bio2/ bio7) (* 100)	/

	bio6	Min temperature of the coldest month	℃
	bio7	Temperature Annual Range (bio5- bio6)	℃
	bio11	Mean Temperature of Coldest Quarter	℃
	bio12	Annual precipitation	mm
	bio13	Precipitation of Wettest Month	mm
	bio15	Precipitation Seasonality (Coefficient of Variation)	/
Soil	t-usda	Topsoil USDA Texture Classification	/
	t-clay	Topsoil clay fraction	%
	t-silt	Topsoil Silt Fraction	kg/d %
	t-pH	Topsoil pH (H2O)	/
Topographical	elevation	Elevation	m
	slope	/	°
	aspect	/	rad
